# Comparing Plan Recognition Algorithms Through Standard Plan Libraries

**DOI:** 10.3389/frai.2021.732177

**Published:** 2022-01-06

**Authors:** Reuth Mirsky, Ran Galun, Kobi Gal, Gal Kaminka

**Affiliations:** ^1^ Department of Computer Science, The University of Texas at Austin, Austin, TX, United States; ^2^ Department of Software and Information Systems Engineering, Ben Gurion University, Be’er Sheva, Israel; ^3^ School of Informatics, University of Edinburgh, Edinburgh, United Kingdom; ^4^ Department of Computer Science, Bar Ilan University, Ramat Gan, Israel

**Keywords:** plan recognition, standardization, plan libraries, theory of mind, artificial intelligence

## Abstract

Plan recognition deals with reasoning about the goals and execution process of an actor, given observations of its actions. It is one of the fundamental problems of AI, applicable to many domains, from user interfaces to cyber-security. Despite the prevalence of these approaches, they lack a standard representation, and have not been compared using a common testbed. This paper provides a first step towards bridging this gap by providing a standard plan library representation that can be used by hierarchical, discrete-space plan recognition and evaluation criteria to consider when comparing plan recognition algorithms. This representation is comprehensive enough to describe a variety of known plan recognition problems and can be easily used by existing algorithms in this class. We use this common representation to thoroughly compare two known approaches, represented by two algorithms, SBR and Probabilistic Hostile Agent Task Tracker (PHATT). We provide meaningful insights about the differences and abilities of these algorithms, and evaluate these insights both theoretically and empirically. We show a tradeoff between expressiveness and efficiency: SBR is usually superior to PHATT in terms of computation time and space, but at the expense of functionality and representational compactness. We also show how different properties of the plan library affect the complexity of the recognition process, regardless of the concrete algorithm used. Lastly, we show how these insights can be used to form a new algorithm that outperforms existing approaches both in terms of expressiveness and efficiency.

## 1 Introduction

A plan recognition algorithm allows an observer to reason about the goals and execution process of an agent, the actor, given a set of its observed actions. It outputs either a sequence of future steps or a hierarchical plan (Schmidt et al., 1978; Kautz, 1987; Bui H., 2003; Blaylock and Allen, 2006; Wiseman and Shieber, 2014; Chakraborti et al., 2017). Recent advancements have applied plan recognition to a variety of real-world domains, including education (Amir and Gal, 2013; Uzan et al., 2015), cyber security (Geib and Goldman, 2001; Bisson et al., 2011; Mirsky et al., 2017b) and more (Masters and Sardina, 2017; Vered and Kaminka, 2017). Although all of these domains have a lot in common in terms of the problem being solved and the components of a recognition problem, there is no single standard representation that allows for a comparison of these works, as they use different models to represent the possible plans an actor can take in the environment (Carberry, 2001; Sukthankar et al., 2014; Mirsky et al., 2021).

This work highlights the lack of standardization in the plan recognition community and proposes a first step towards defragmentation of this community, by presenting a standardized representation that allows several algorithms and domains to be evaluated using similar metrics. We create a joint interface that facilitates two representative plan recognition algorithms from the literature, and then use this representation in order to provide a thorough, both theoretical and empirical, comparison between them. This comparison then enables us to leverage insights from these works and provide a new, improved algorithm that outperforms both baseline algorithms.

This work presents the following contributions:

1) A general plan library representation that can be used as a baseline representation for plan recognition contributions to refer to.
2) A theoretical and empirical comparison of two representative plan-library based plan recognition algorithms (SBR and PHATT, see below).
3) Leveraging the insights learned to provide an improved PHATT algorithm.

The choice to use plan-libraries for plan recognition stems from their high expressiveness, and their ability to provide robust explanations without any demonstrations or data sampling (Kim et al., 2018; Kantharaju et al., 2019). Within the scope of plan-library based plan recognition, there is a variety of algorithms and representations, including AND/OR trees, grammars, Hierarchical Task Networks (HTNs) (Erol et al., 1995), Temporal Plan Networks (TPNs) Kim et al. (2001), and more. We focus here on two families of algorithms: graph-based algorithms that explicitly depict the full set of potential plans; and grammar-based algorithms that capture the fragments of these plans and how they can be combined.

For our comparison, we will use two plan recognition algorithms from these two families: the graph-based SBR (Avrahami-Zilberbrand and Kaminka, 2005) and the grammar-based PHATT (Geib and Goldman, 2009). PHATT and SBR were both designed to perform plan recognition using a plan library, but are fundamentally different. As a grammar-based algorithm, the PHATT algorithm was inspired by language parsing. The graph-based SBR algorithm, on the other hand, was inspired by hierarchical plan representations and robotics and its focus is on fast performance.

The differences between the approaches translate into different abilities of the algorithms: SBR is faster in its on-line processing and can give partial answers about the current state of the actor, but these answers might not be correct once more observations are revealed (Avrahami-Zilberbrand and Kaminka, 2005). PHATT (Geib and Goldman, 2009), on the other hand, is more comprehensive, can handle more complex inputs and outputs, but its runtime is sometimes even exponentially slower than SBR, as we show in this paper.

This work provides a first thorough evaluation of plan-library based plan recognition, raising several interesting insights, both specific to the algorithms evaluated and to plan recognition research in general. In the next section, we present some basic concepts and plan recognition background. Then, we present the compared algorithms and outline the approach each is using. Section 2 discusses related work. Next, Section 4, describes the proposed standard representation for plan-library based plan recognition, and lists the properties it can capture. Then we highlight the differences between the compared algorithms and consequently, and the constraints needed to be enforced to enable a fair comparison of the algorithms. This section concludes with a theoretical complexity analysis of the two algorithms. Section 5 presents a comprehensive empirical evaluation of the two algorithms, using an artificially generated domain in which various important plan recognition parameters can be modified. Then, Section 6 uses the lessons and insights learned from this evaluation to leverage improvements and work done on SBR to improve PHATT. Finally, we conclude and provide a list of guidelines for future works in Section 7.

## 2 Related Work

As plan recognition plays a crucial role in a variety of applications and disciplines, researchers have a diverse set of techniques and representations when approaching a plan recognition problem, depending on their application domain. This fragmentation causes to a lack of ability to compare between the different algorithms, as each is aimed to optimize a different set of requirements (Carberry, 2001; Sukthankar et al., 2014; Mirsky et al., 2021).

Many plan recognition algorithms use plan libraries to represent the possible plans of an actor in the environment. These plan libraries are highly expressive and their explicit representation of plans makes them inherently explainable. But while this expressive richness enables it to be used in a variety of domains including education, robotics, e-commerce, and verification, it also leads to the use of different models to represent the possible plans an actor can take in the environment. To this date, there is no commonly accepted standard representation that allows for a comparison of these works (Mirsky et al., 2018).

### 2.1 Plan-Library Based Plan Recognition Algorithms

There are several approaches to represent a domain in plan recognition. Some recent advent of work on plan recognition as planning takes as input a planning domain, usually described in STRIPS (Standford Research Institute Problem Solver) (Fikes and Nilsson, 1971), a set of possible goals and selects one of the goals (Ramırez and Geffner, 2010; Sohrabi et al., 2016; Freedman and Zilberstein, 2017; Masters and Sardina, 2017; Pereira et al., 2017; Shvo et al., 2017; Vered and Kaminka, 2017). In this work, we focus on PL-based plan recognition (Blaylock and Allen, 2006; Sukthankar and Sycara, 2008; Kabanza et al., 2013; Geib, 2017; Barták et al., 2018). We consider plan library representations that are less expressive from or equivalent to a hierarchical task networks (HTN) Erol et al. (1995), but that do not take into account pre and post conditions. Previous work in this class of recognizers used PHATT or SBR as a baseline to compare against, depending on the representation that was used for the PL, and showed aggregated improvements based on specific properties.

Yet Another Probabilistic Plan Recognizer [YAPPR, (Geib et al., 2008)] can improve PHATT’s runtime significantly if the user is not interested in complete plans, but rather in the goals of the actor and predictions about future actions. The Decision-Oriented PLAn Recognizer [DOPLAR, (Kabanza et al., 2013)] extended YAPPR using probabilistic reasoning to reach even better performance, at the cost of completeness. Cumulative Recognition of Activities and Decreasing Load of Explanations [CRADLE, (Mirsky et al., 2017a)] augmented PHATT with the ability to process PLs with parameters and proposed a set of pruning heuristics.

Avrahami-Zilberbrand and Kaminka (2005) enhanced the basic SBR to handle interleaving of more than one plan in Avrahami-Zilberbrand et al. (2005), and proposed an algorithm that reasons about the utility of the actor as part of the recognition process (Avrahami-Zilberbrand and Kaminka, 2007). Sukthankar and Sycara (2008) based their work on SBR to deal with large domains and multiple agents.

Other notable works are Engine for LEXicalized Intent Recognition [ELEXIR, (Geib, 2009)] and YR (Maraist, 2017) which present new approaches for PL-based plan recognition algorithms. These algorithms use PL representations that differ both from PHATT’s and from SBR’s. Another possible standard representation that was considered in previous work is the Hierarchical Task Network (HTN) representation used by the Simple Hierarchical Ordered Planner 2 (SHOP2) (Nau et al., 2003). However, this is a state-based representation, which means that the goal of an agent is a state (or a set of predicates), while in the algorithms presented in this paper, the goal is a complex action (or a set of such). This makes the use of these domains to be non-trivial, as it is not straightforward converting from a goal state to a set of goal actions.

All of the above papers used a single existing algorithm as a baseline and showed improvements in specific properties, but they did not compare to more than one algorithm nor did they investigate the differences between the algorithms.

### 2.2 Standardization

Previous papers surveyed methods for plan recognition, but did not try to run and evaluate the presented algorithms as well (Carberry, 2001; Sukthankar et al., 2014; Albrecht and Stone, 2017; Mirsky et al., 2021; Van-Horenbeke and Peer, 2021). Many of these surveys discuss the research efforts in plan, activity and intent recognition (PAIR) and present open challenges that are relevant to the PAIR community, such as: 1) designing computationally efficient algorithms, and 2) finding a single representation that can naturally generate various modelling capabilities. The work presented here is aimed to provide a first step towards meeting these challenges.

## 3 Background: PHATT and SBR

This section provides the necessary background for representing a plan recognition domain and using PHATT and SBR. First, we identify the components of a plan recognition problem in the scope of this work. We used standard definitions from the plan recognition literature, in which basic actions represent rudimentary activities that cannot be decomposed, and complex actions represent higher level activities that can be decomposed to basic and complex actions (Gal et al., 2012). To emphasize the distinction between these two action types, we notate basic actions using the **bold** notation.

An *observation sequence* is an ordered set of basic actions carried out by the acting agent. A *plan library* is an explicit yet compact representation of all possible plans that an actor can execute in the environment. These plans can be represented using graphs, AND/OR trees, or grammars. As SBR and PHATT use different representations for their plan libraries, we will provide a separate definition of a plan library for each. Given an observations sequence and a plan library, the goal of the *observer* is to infer the plan of the *actor*.

We will use a running example based on a real-world domain, an open-ended educational system called TinkerPlots. It is used world-wide to teach students in grades four through eight about statistics (Konold and Miller, 2004). Using TinkerPlots, students build stochastic models and generate pseudo-random samples to analyze the underlying probability distributions. Several prior works have used plan recognition to infer students’ activities in such open-ended environments (Conati et al., 1997; Amershi and Conati, 2006; Amir and Gal, 2013; Mirsky et al., 2017a). We will use one of the problems given to students (ROSA) in our running example:

There are 4 letters printed on cards, each card contains one letter: A, O, R, S. The cards are lined up in a row. After mixing the cards up, what is the probability that the cards would spell ROSA?

In order to solve this problem, a student must perform the following activities using the software: create a sampler model of a probability distribution over ROSA; run the model and collect data; plot the results correctly on the graph. When accomplishing all activities successfully, the student is said to have solved the ROSA problem. The basic level actions in the plan library for solving the ROSA problem are the actions that students can perform in the software, such as add new sampler (**NS**), Create device (**CCD**), which is executed by adding a device to sampler (**SAD**), set number of draws in the sampler (**SDS**) and number of repetitions (**SR**); *SRP*, *CSM* and *PO* are some of the complex actions.

For example, the complex action of solving the ROSA problem (*SRP*) is decomposed into the following activities: creating a sampler object (*CSM*), running the sampler (**R**) and plotting the sampler on a graph (*PO*). **R** must follow *CSM*, as one must first create a sampler before it can be used to run tests. *PO*, creating a plot, can be executed only after **R**. Recipes describe these relationships and how complex actions decompose into other complex and basic actions, and in what order.

Both algorithms represent the possible activities that can be carried out by the student. However, their representation differ very much.

### 3.1 The PHATT Representation

We begin by describing the representation used by the PHATT algorithm.

Definition 1 (Plan Library in PHATT) A plan library (PL) is a tuple *L* = ⟨*B*, *C*, *G*, *R*⟩, where *B* is a set of basic actions, *C* is a set of complex actions, *G* ⊆ *C* is the set of goals the actor can achieve and *R* is a set of recipes, such that each recipe is of the form *c* → (*τ*, *O*), where (1) *c* ∈ *C*; (2) *τ* ∈ (*B* ∪ *C*)*; (3) and *O* is a partial order over *τ* representing ordering constraints over the actions in *τ*.

**FIGURE 1:**
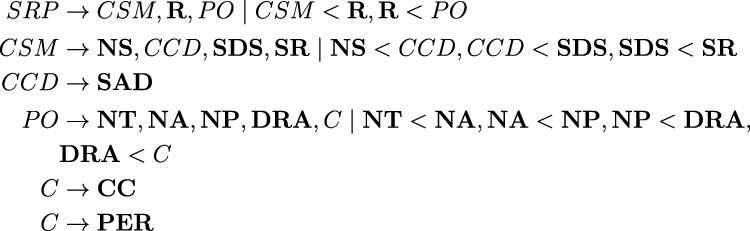
PHATT PL for the ROSA problem.

Figure 1 is an example PL for the ROSA problem. Each line represents how a complex action constitutes a partially ordered sequence of basic and complex actions. For example, the first recipe defines the action *SRP* (Solve ROSA Problem) to be constructed from *CSM* (Create Sampler), **R** (Run Sampler), and *PO* (Plot the sampler to a graph).

A *plan* in a PL *L* is a labeled tree p=(V,E,L) , where 1) *V* and *E* are the nodes and edges of the tree, respectively, 2) L is a labeling function L:V→B∪C mapping every node in the tree to either a basic or a complex action in *L*, and 3) the children of a node labeled by a complex action are a decomposition of this complex action into constituent actions, according to one of the recipes. The set of all leaves of a plan *p* is denoted by *leaves*(*p*). Note that sibling nodes in a plan can have implicit ordering constraints between them, according to the recipe used to create them. A plan is said to be *complete* iff all its leaf nodes are labeled basic actions, i.e., ∀v∈leaves(p),L(v)∈B .

An *observation sequence* is an ordered set of basic actions that represents actions carried out by the observed agent. A plan *p* *describes* an observation sequence *O* iff every observation is mapped to a leaf in the tree. As we will see next, in the SBR representation, an observation sequence can be an ordered set of both basic and complex actions.

As mentioned earlier, the PHATT algorithm was inspired by parsing techniques, where the recipes are given in the form of a grammar, observations can be considered words, and each outputted plan is a parse tree. The major problem with parsing as a model of plan recognition is that it does not treat partially-ordered plans or interleaved plans well. This situation resembles to a case of parsing two (or more) sentences that their words are mixed together. Both partial ordering and interleaving of plans result in an exponential increase in the size of the required grammar, issues which have been addressed in the implementation of PHATT (Geib and Goldman, 2009).

**FIGURE 2:**
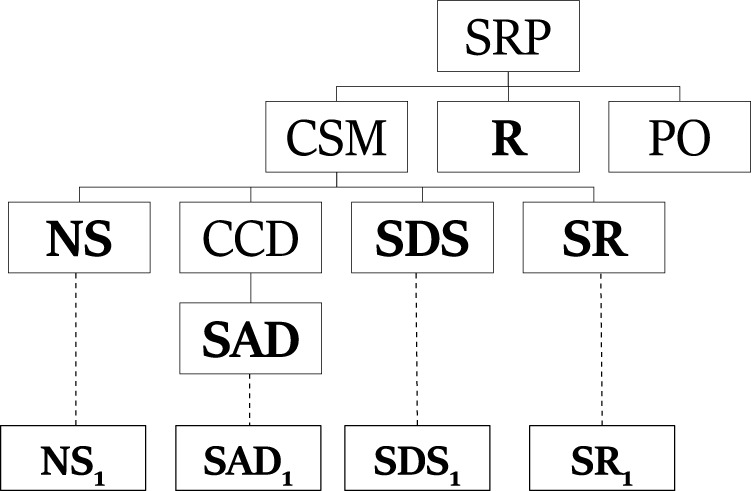
A plan describing the observation sequence **NS**, **SAD**, **SDS**, **SR** in PHATT-style.

Figure 2 shows a plan that describes the observation sequence **NS**, **SAD**, **SDS**, **SR**. In a PHATT-style plans, the goal of the plan, *SRP*, is the root node and the student started executing the basic actions, mapped to leaves in the plan, to achieve *CSM*. This plan is an *incomplete plan* explaining the student’s actions. Incomplete plans include nodes labeled with complex level actions that have not been decomposed using a recipe (like *PO*) or basic actions which has not been mapped to an observation (like **R**). These *open frontier* nodes represent activities that the agent will carry out in future. Finally, an *explanation* that describes an observation sequence is a set of plans such that each plan describes a mutually exclusive subset of the observation sequence and taken together the plans describe all of the observations.

We now provide here a brief description of the PHATT algorithm. At the first observation *σ*, PHATT constructs explanations describing this observation. Each explanation includes a single plan tree such that 1) the root node is labeled with a goal *g* ∈ *G*; 2) there exists a leaf node that is labeled with *σ*; 3) the path from the root to the leaf includes actions that are derived from the recipes of the PL; 4) all other leaves in the plan tree are open frontier nodes, and the plan is referred to as a *leftmost tree deriving* *σ*. For a complex action *C*, the *generating set of* *C* is the set of all leftmost trees that derive some basic action *σ*, that their root is labeled with *C*.

When introducing a new observation *σ*_*n*_ into an existing explanation, PHATT considers two possibilities:

1) Adding *σ*_*n*_ as a new plan in the explanation, which requires to iterate the generating sets of all *g* ∈ *G* to find the leftmost trees that derive *σ*_*n*_.
2) Updating an existing plan in the explanation with *σ*_*n*_. This option requires to construct another leftmost tree deriving *σ*_*n*_ with a root that matches one of the open frontier nodes of an existing plan.

This process is iterated over all observations, maintaining a set of explanations *H* that explain the sequence of observations seen so far. To this end, PHATT modifies and extends the explanations in *H*, and, if necessary, adds and removes explanations, so that *H* contains explanations that describe the new updated sequence of observations *σ*_1_ … , *σ*_*n*_.

**FIGURE 3:**
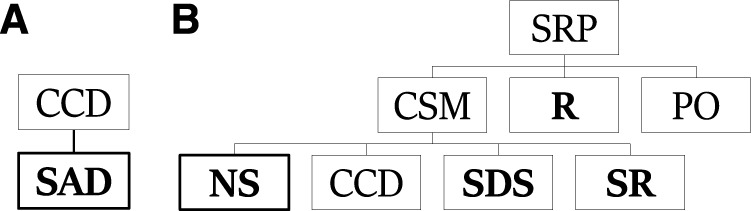
Leftmost deriving trees for *SRP* **(B)** and *CCD* **(A)**.

Figure 3 exemplify components from PHATT’s explanation construction process. The tree on the right is one tree from the generating set for *SRP*. It is a leftmost deriving tree, which derives **NS**. The tree on the left is such a leftmost deriving tree for **SAD**, and a part of the generating set for *CCD*.

**FIGURE 4:**
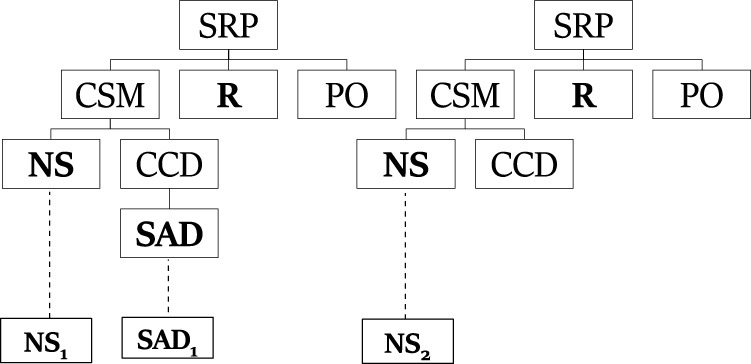
An explanation created by PHATT for the ROSA problem.

Assume the following observation sequence: **NS**_**1**_, **SAD**_**1**_, **NS**_**2**_ is given. This sequence means that a student created two samplers and added a device to the first sampler. Using the trees from the generating set and this observation sequence, Figure 4 shows one explanation that PHATT will output, where the first and third observations are described by the left plan, and the second observation is described by the right plan.

### 3.2 The SBR Representation

Definition 2 (Plan Library in SBR) is a single-root directed acyclic connected graph, where vertices denote plan steps, and edges can be of two types: vertical edges (also called decomposition edges) decompose plan steps into sub-steps, and sequential edges specify the expected temporal order of execution.

In the original SBR paper (Avrahami-Zilberbrand and Kaminka, 2005), a PL has a single root node. The set of possible goals (top-level plans) were defined as the children of the root node, and all other nodes are plan steps. In this formalism, similar to the PHATT formalism, we refer to basic actions as leaves in the graph, and complex actions to all intermediate nodes.

**FIGURE 5:**
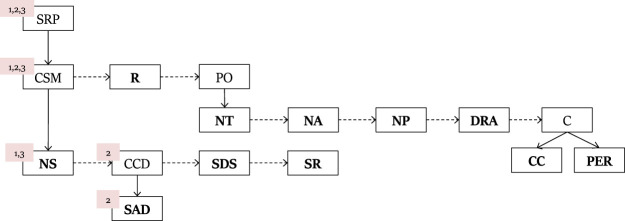
The PL structure created and used by SBR for the ROSA problem.

Figure 5 shows the SBR PL constructed for the ROSA problem. In this representation, a dashed arrow from node *A* to *B* means that the action *A* must precede *B*. A full arrow from *A* to *B* means that *B* can be the first action in a sequence of actions that compose *A*. This figure represents the basic structure that SBR builds to store all possible plans. For example, the actions the student needs to execute to perform a plotting action (*PO*) are ordered, so they appear using dashed lines. A complex action and its first constituent action are connected using a full line. Setting the plot type to count can be executed using a case count visualization (**CC**) or by percent (**PER**), so these to options appear as choices under *C* using a full line.

Since in the SBR algorithm the plan library is a single tree that represents all possible plans, an SBR *plan* for achieving some action *A* is a path along the plan library tree from a node labeled with *A* to the root. This plan is represented by marking each node in the path from the root to *A* with a timestep.

**FIGURE 6:**
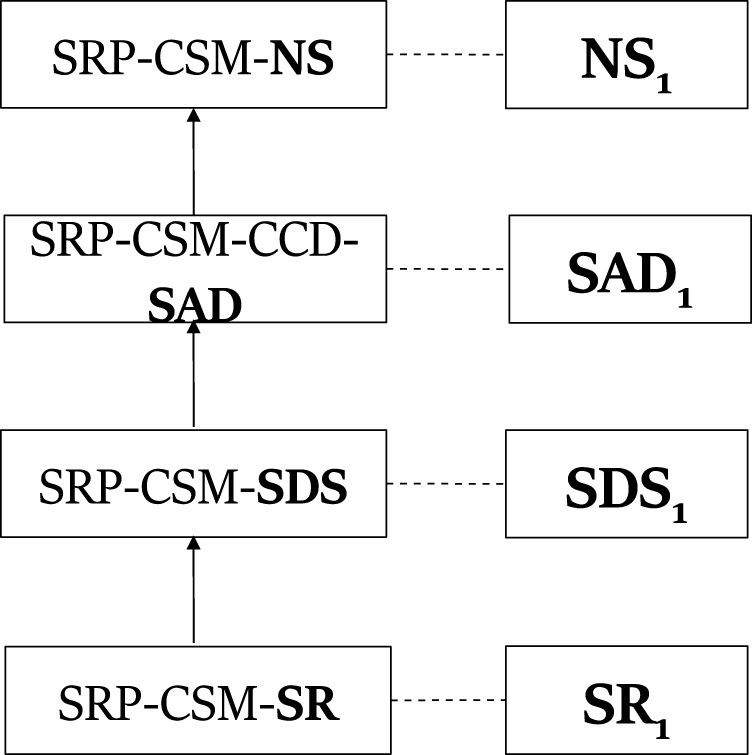
A plan describing the observation sequence **NS**, **SAD**, **SDS**, **SR** in SBR-style.

A plan *p* *describes* an observation sequence *O* iff every observation *O*_*i*_ marks a path from the root to a node (rather than to a leaf in PHATT) in the plan library such that each node on that path is marked with the timestep *i*. This marking is done in a consistent manner, meaning that some node *X* cannot be labeled with a timestamp *t* unless it is a first step in a new plan, or it has an incoming sequential edge from a node with a timestamp *t* − 1. Notice that in a valid plan the describes *O*_1_, … , *O*_*n*_, the root should be marked with all timesteps 1, … , *n*. Figure 5 illustrates how a plan describing the observation sequence **NS**, **SAD**, **SDS**, **SR** is marked on top of the plan library structure. Consider the first observation **NS**: to reach the node labeled with **NS** from the root, one must go through a node labeled with *SRP* ∈ *G*, then to its child node labeled *CSM*, and finally to the node labeled **NS**. This means that all nodes **NS**, **CSM**, **SRP** should be marked with timestep 1. Figure 6 presents a compilation of these markings into a consistent set of paths from the root to each observation, and the mapping between these paths (on the left of the figure) to the observations (on the right).

Agents are assumed to plan by choosing a subset of complex actions as intended goals and then carrying out a separate plan for completing each of these goals, using basic or complex actions (unlike PHATT, where the agents can only be observed executing basic actions).

As in PHATT, an *explanation* that describes an observation sequence is a set of plans such that each plan describes a mutually exclusive subset of the observation sequence and taken together the plans describe all of the observations.

Given an observation, SBR traverses the PL and labels specific nodes upon it that can represent the observation with a timestamp, in a way that is consistent with only the last observation. At this point, SBR can provide simple feedback about what can the current step that the agent executed. This feedback is called Current State Query (CSQ).

After a sequence of observations *O*_1_, … , *O*_*n*_ were marked on the PL, SBR can also be requested to output the plans describing them. It traverses the structure once more and collects consistent paths that can explain the observation sequence soundly, in a process called History State Query (HSQ*)* (Avrahami-Zilberbrand and Kaminka, 2005). This process is done by retracing the root-to-observation paths that were travered by SBR, and merging the repeating nodes. For example, in Figure 6, the *SRP* − *CSM* − **NS** path that leads to the observation **(*NS*)** and the path *SRP* − *CSM* − *CCD* − **SAD** that leads to the observation **SAD** can be merged into one explanation, where *SPR* is the root of the explanation, *CSM* is its child, and *CCD* and **NS** are the two children of *CSM*.

Assume the same following observation sequence as before: **NS**_**1**_, **SAD**_**1**_, **NS**_**2**_. SBR will construct the initial PL from Figure 5 once in the initialization stage, and every time it will process an observation, it will mark its timestamp on that single structure. Thus, the **NS** node will be labeled with timestamps 1 and 3, while the node **SAD** will receive the timestamp 2. At this point, the CSQ process can return a feedback in the form of “The actor can currently be at node **NS** on the path to *SRP* through *CSM*”.

**FIGURE 7:**
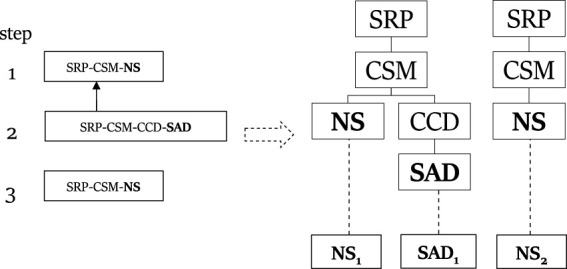
An extraction graph created by SBR and its translation to an explanation.

If the HSQ process is evoked, it will construct a secondary data structure, called *extraction graph* as depicted in Figure 7(left). This graph captures that there is a continuity between the first and second steps, such that the **SAD** action comes after the first **NS** action. The second **NS** action is independent. Using this extraction graph, the resulting explanation (Figure 7(right) can easily be constructed by traversal over the created paths in the extraction graph.

## 4 Comparative Theoretical Analysis

SBR and PHATT were chosen for this evaluation since their input and output can be easily standardized. However, there are major differences between the algorithms before which makes their comparison challenging. This section highlights these differences between the algorithms and describe how to account for them in the analysis.

**FIGURE 8:**
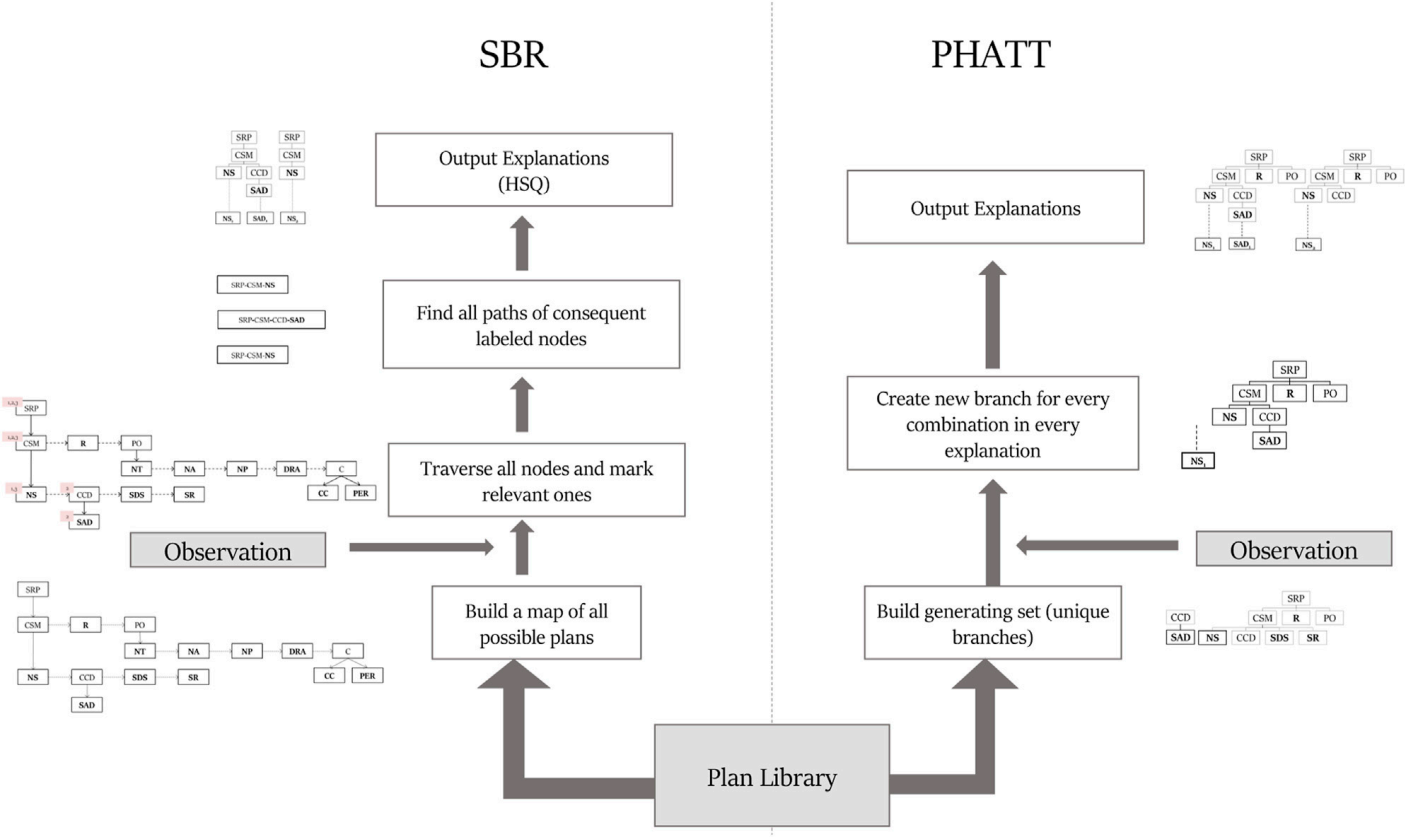
Depiction of the flow differences between SBR and PHATT.

Figure 8 depicts the main flow of both algorithms as detailed below and summarizes the different data structures used in these algorithms. A summary of the differences between the algorithms appears below, and discussed in detail in the following sections:

1) Recursion: PHATT allows recursive recipes that SBR does not (e.g., X → **Y**X, X → **X** and a plan sequence **Y**, … , **Y**, **X**). Discussed in Section 4.1.
2) Interleaving: PHATT can handle plan interleaving (e.g., the explanation in Figure 4 can be a valid recognition hypothesis for the observation sequence **NS**_**1**_, **NS**_**2**_, **SAD**). Discussed in Section 4.2.
3) Current State Query: SBR can provide intermediate “current state queries” (CSQ). Discussed in Section 4.3.
4) Observing Complex Actions: SBR can accept complex actions as observations. Discussed in Section 4.3.
5) Theoretical Complexity: PHATT and SBR are constructed differently, and hence their use of space and time is different. Discussed in Section 4.4.

### 4.1 Bounded Recursion

To be able to discuss the similarities and differences of the two algorithms, and to show that PLs for PHATT and SBR can describe the same space of plans, there is a need to explain how recursion can be used in PLs. Recursion in a PHATT PL means that a recipe can have an action as a constituent of itself. Recursion in an SBR PL means that there is a decomposition edge from a node labeled *A* to either 1) another node that has the same label *A*, or 2) there is a sequential node that follows the decomposition with the same label *A* as the origin node. Bounded recursion in both of these representations means that these recursions can only be used a predefined number of times.

Claim 1. Assuming that the use of recursion is bounded, the PLs used by PHATT and SBR have the same expressibility.

Proof. We first show how a PHATT PL can be translated into an SBR PL. For each goal action G in PHATT-style PL, we construct a node in SBR below the root. Then, recursively, for each recipe r: c → (τ, O) such that there is already a node labeled with c in SBR, for every possible permutation of the constituents in the recipe (according to O), we add |τ| nodes below c and label them with respect to the permutation. Since we assume bounded recursion, at some point all leaves in the SBR PL will be either basic actions or recursive complex actions that was bounded by the recursion bound assumption.

Next, we show how an SBR PL can be translated into a PHATT PL. According the definition of a PL in SBR, we define G to be the set of all nodes below the root node. The label of a node with no decomposition edges leaving it is a basic action in B. All other intermediate nodes represent a complex in PHATT, as part of the set of complex actions C. Notice that if two nodes in SBR have the same label, this means that an action is used in two different places in the SBR PL (e.g., used in two different recipes of PHATT). For each decomposition edge from a node labeled A to a node labeled B, we define a recipe as: A → Bτ∣O, such that τ is the ordered sequence of nodes that are connected to B sequentially, and O is the order of these nodes according to the sequential edges. Notice that using this translation, the recipes created are always fully ordered.

### 4.2 Completeness

Both SBR and PHATT are considered complete algorithms, but this notion of “completeness” is different for each algorithm due to differences in their underlying assumptions: some fundamental properties cause these algorithms to output different explanations given the same input. The goal of this subsection is to evaluate these differences and constrain the PL such that the algorithms will be evaluated when they output exactly the same explanations.

Consider the full TinkerPlots PL, which has the following properties: its maximal And-branching factor is 5, maximal Or-branching factor is 2, an alphabet size of 32 and a maximal depth of 3. This PL is highly recursive, and the recipes can create an unbounded number of permutations of plans. While PHATT has the ability to create plans of unlimited depth, SBR cannot output plans that were not created during initialization. Due to this difference, the algorithms output a very different set of explanations: for a sequence of mere 4 observations, SBR outputted 2 explanations, while PHATT outputted 6000 (with a recursion bound of 3).

Another difference is that PHATT has the ability to handle interleaved plans, while the original SBR algorithm does not Avrahami-Zilberbrand and Kaminka (2005). Both algorithms can reason about an actor that executes more than one plan, but only PHATT allows the actions of the plans to interleave.

For example, when cooking a dinner, the actor might make a pasta dish and a salad dish. Suppose the first action observed is to start boiling water for the pasta. The next action might be chopping vegetables for the salad. In such a scenario, PHATT will output more explanations than SBR, as it will also show ones in which several plans interleave.

To reason about interleaved plans, PHATT is required to keep track of all unfinished plans, which means it keeps a separate copy of every plan in every explanation rather than using a single representation covering all possible plans. SBR was later extended to account for interleaved plans Avrahami-Zilberbrand et al. (2005), but this is not the version we utilized in this paper.

Even when the PL representation do not explicitly allow interleaved plans, the algorithm can receive observation sequences that can be described by such plans. In the following example, based on the presented TinkerPlots domain, a given observation sequence can be: **NS**_**1**_, **NS**_**2**_, **SAD**_**1**_. This means that a student created two samplers and only then added a device to the first sampler. Notice that this is a different sequence than the **NS**_**1**_, **SAD**_**1**_, **NS**_**2**_ sequence used earlier, which does not contain interleaved execution of actions from different plans. Figure 4 depicts one of the explanations that can be outputted from PHATT for this observation sequence. The left plan is incomplete when the **NS**_**2**_ is executed, and then we come back to it with the action **SAD**_**1**_. PHATT outputs this explanation (among others), while SBR does not output it. Other than that, all other explanations appear both in PHATT and SBR’s outputs.

Notice that this difference is only apparent when plans interleave - if one plan is complete and only then another begins, or if we don’t return to the first plan after starting the second one - there is no difference between the algorithms. However, in our empirical evaluation, an instance with such interleaving only appeared 62 times in 1091 runs, mostly in PLs with increased depth. In two of these cases, for example, SBR outputted 3 and 4 explanations. PHATT outputted 4 and 5 explanations, respectively. In these cases, the additional explanation had two plans with interleaved actions. Thus, we excluded these specific scenarios from the experiments reported below.

### 4.3 Algorithm-Specific Properties

Another major difference between these two algorithms is SBR’s inherent capability to provide an answer to a query about the actor’s current state (Current State Query, or CSQ), without the need to reason back through all of the previous observations. For example, in the TinkerPlots domain, we might want to understand whether the student is currently working on the ROSA problem, or performing an action that biases from any solution. In such a scenario, we might like to alert the teacher that the student is doing something wrong, regardless of their previous actions.

The ability to output only the current state gives the algorithm real-time responsiveness, but at the expense of consistency with past observations: a sequence of returned current states (i.e., the sequence of CSQ answers) might not be completely consistent with the plan library, since given observations and appropriate recognition hypotheses at a time t, it might be possible to now rule out explanations that might have been correct at an earlier time t′, where t′ < t. To find fully consistent explanations for the entire sequence of observations, SBR relies on a separate algorithm for computing the actors history of states (HSQ). PHATT, on the other hand, computes the full set of explanations for all observations, with every observation. It can report on the current state only by computing complete explanations and then eliciting the current possible states. As we show below, the separation of queries reduces the complexity of recognition for SBR.

An additional property of SBR is that it can accept both basic and complex actions as inputs, and use them to generate recognition hypotheses. PHATT can only take basic actions as input. Extending PHATT to reason about complex actions as well is a trivial change in the code, but it was not part of the original algorithm. However, this ability was added in a later work (Mirsky and Gal, 2016). In this work, however, as we compare the vanilla versions of the algorithms, we do not use complex actions as observations as part of the empirical evaluation.

There are additional properties that both algorithms keep, and every comparable algorithm (such as Geib (2009) or Kabanza et al. (2013)) will have to keep as well: Both algorithms can handle partial ordering of the actions, which is a compact way to represent a sequence of actions that can be performed in several permutations. Both can output the complete plans of the actor rather than just their goals. Both algorithms can also reason about observation sequences that execute more than one plan, although as described above, only PHATT allows these plans to interleave.

### 4.4 Algorithm Complexity

Plan recognition in exploratory domains using plan grammars, similar to the ones used in PHATT, is known to be NP-hard (in general), as shown by (Gal et al., 2012) who presented a reduction of this problem to a constraint-satisfaction problem (CSP). In this section, we drill down into the pseudo code of the algorithms in order to map the critical parts of each algorithm in terms of time and space complexity.

Algorithm 1 The PHATT algorithm

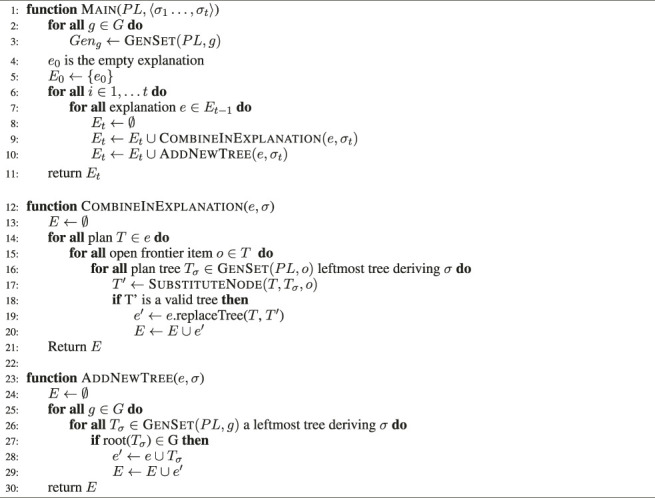

The pseudo-code of PHATT is presented in **Algorithm 1**. The initialization of PHATT takes place in lines 2–5: as discussed in Section 3, the generating set for complex action c is the set of branches with a single basic action in the leaf-level and c as the root. The size of the generating set for a single action is bounded by the maximal or-branching of the PL (denoted MaxOr) power by the maximal depth of the PL (denoted MaxD). In total, the generating set for the complete PL is expected to be |G|× MaxOr^MaxD^. More details on the construction of the generating set can be found in Geib and Goldman (2009).

After the initialization, the algorithm incrementally creates explanations for the observations. It checks if a new observation can be combined into the existing explanations either by adding a branch from the generating set as a substitute to a leaf in an existing tree, or as a new tree.

The function SubstituteNodeT, T_σ_, o is a function that takes a tree T with a leaf node labeled with o, a subtree T_σ_, and the specific instance of o (in case there is more than one leaf labeled with o). It returns an updated tree T′, which is T with T_σ_ instead of the node labeled with o. The complexity of this function is O(1). However, considering all the possible points of substitution for an observation σ_n_ in an explanation e is O(MaxAnd^MaxD^) where MaxAnd is the maximal and-branching of the PL. This process needs to be done for every explanation, so for the nth observation, this needs to be done O(|E_n−1_|× MaxAnd^MaxD^) times. After the substitution, a new copy of the new explanation with the new observation needs to be kept. This is the part that is the most time- and space-consuming in the PHATT algorithm.

The pseudo-code of SBR is presented in **Algorithm 2** [see Avrahami-Zilberbrand and Kaminka (2005)]. It is separated into two main functions, one for the current state query (CSQ, at line 1) and the other of the history state query (HSQ, at line 16). The details of specific calls is discussed below.

The algorithm starts with initPLPL (line 2), which is the function that takes a PL and constructs all possible plans. This process is a hotspot of the SBR algorithm, and it creates O(MaxAnd × MaxOr^MaxD^) nodes, exactly once.

Then, for each observation σ_i_, the function FindNodeP, σ_i_ traverses the PL and returns all instances of σ_i_ in the PL. Naively, this is a search over all nodes, but Avrahami-Zilberbrand and Kaminka (2005) describes a method for carrying this out at a complexity of O(MaxD). For each node n, a node that represents an instance of σ_i_, isRootn returns true iff n is a root node in the PL, and IsConsistentn returns true iff n appears in the PL as a first child of a plan or as the next sibling of a previously tagged node. If these two conditions are met, AddTagn, σ_i_ adds a tag of the current time (number of observation = i) to the node n. tagged is the list of all tagged nodes so far. All of these steps are carried out in O(1). Finally, getPathsΠ, p_1_, p_t_ returns all paths in Π on tagged nodes such that p_i_ is tagged with i for all i ∈ 1‥t and each node p_i_ is consistent with respect to the previous nodes. Overall, the generation of the SBR PL is bounded by O(MaxAnd × MaxOr^MaxD^), and the tagging process traverses this PL once per observation, where each consistency check to see if a node can be tagged is carried out in O(1). Together these operations make the CSQ process bounded by O(MaxAnd × MaxOr^MaxD^ + n). The HSQ process just require to traverse the nodes that were already decided to be consistent, it will need to travese, in the worst case, the whole plan library once. Thus, the final expression for the execution complexity of SBR is O(MaxAnd × MaxOr^MaxD^ + n).

## 5 Empirical Comparison

In this section we present several empirical results that exemplify how the different properties discussed above affect the runtime and space usage of PHATT and SBR. We begin by describing the properties of the domains tested, and how they were controlled using a domain generator. We then show how each domain property affects each algorithm, and dive into the different components that take place in the recognition process in both algorithms. We then show the completeness results of both algorithms, if we assume that interleaving is allowed.

Algorithm 2 The SBR algorithm

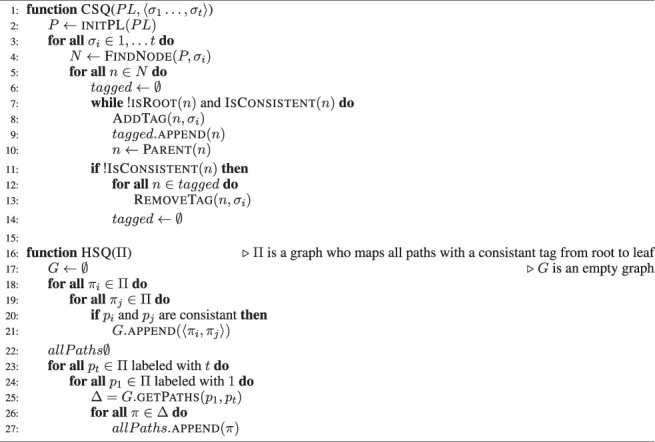

### 5.1 Domain Generator

In order to evaluate the impact of several PL properties, we used the PL generator from Kabanza et al. (2013), that can be configured to output PLs that vary in several features which are known to affect the explanation set size (Geib and Goldman, 2009), but that were never compared on more than one algorithm at a time. The parameters of the PL that can be configured are:

• Number of Goals Representing the number of different goals an actor might pursue at the same time.
• Depth Representing the depth of the PL. Generally in PLs, this value is the depth of the deepest plan that can be created in the library. However, all plans generated on the same call for this generator will have the same depth, so in this evaluation, the Depth value is set to be the depth of all plans.
• Alphabet Size Representing the number of basic actions in the PL.
• Or Branching Factor This is the number of different ways a complex action can be decomposed into a sequence of constituent actions.
• And Branching Factor This is the number of constituents which decompose a complex action.
• Ordering This is the type of ordering constraints that can be put on a sequence of constituent actions. The possible orders are: unrodered, fully-ordered, partially ordered (with a default value such that %30 of the constituents are ordered), first and last (where only the first/last constituent is strict and the others are unordered).

The generator outputs a PL which is constructed from partially ordered AND/OR trees. We then translate these trees to a set of recipes: an AND node is translated into a single recipe, with the same ordering constraints as the partial order of the AND node’s children. An OR node labeled with an action c is translated into a list of recipes with c as their left hand side, such that each recipe represents one option that can be executed to achieve c.

**FIGURE 9:**
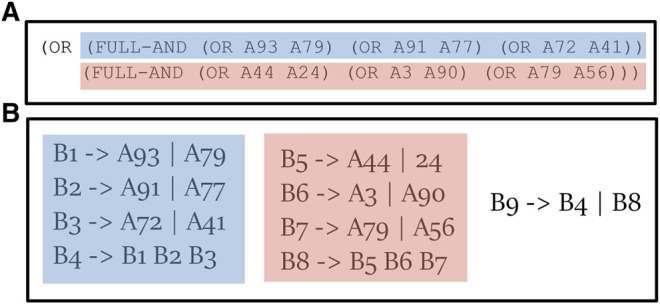
An example of generated plan library **(A)** and its translation to a set of recipes **(B)**.

An example of one of the generated PL can be viewed in Figure 9 (as this is a fully-ordered plan, ordering constrains are omitted for brevity). At the top of the figure, there is a fragment of a plan generated with an AND branching factor of 3, and an OR branching factor of 2. All basic actions are given a number and are labeled with a prefix of **A** (e.g., **A93**). The translation process create a recipe, or a set of recipes, for each operator in this tree (bottom). Each OR node is translated into a list of recipes, for example, (OR **A93 A97**) is translated into two recipes: B1 → **A93** and B1 → **A79**. Each AND node is translated into a single recipe, for example: B4 → B1 B2 B3. Notice that the complex actions were implicit in the AND/OR tree, and become explicit in the translation into recipes.

As a baseline, we used the same configuration as (Kabanza et al., 2013). In the baseline PL, the actions are fully ordered, the number of possible goals is set to be 5, the depth is set to 2, the alphabet size to 100, the AND branching factor is set to 3, and the OR branching factor is set to 2.

In the following experiments, we also varied the depth of the domain (from 1 to 4), the or branching factor (from 1 to 4) and the and branching factor (from 2 to 5). We did not change the number of goals and ordering because they add an additional complexity to the PL that causes PHATT and SBR to behave differently, as discussed in Section 4.2. We did vary the alphabet size in our experiments (to 20 actions instead of 100), but the results where not significantly different from the baseline domain in any aspect, so we do not show them here.

In this section, we present the evaluated runtime of the algorithms on the various PLs. Both algorithms are implemented in Python and are tested on the same commodity i7 computer. It is important to highlight that this is a single implementation of each algorithm, and hence we do not provide direct runtime comparison but rather count basic operations. We also stress that this comparison is only meant to provide with general notion of the algorithms’ abilities, and that the theoretical evaluation of their performance can be found in Section 4.4.

### 5.2 Time and Memory Consumption

**FIGURE 10:**
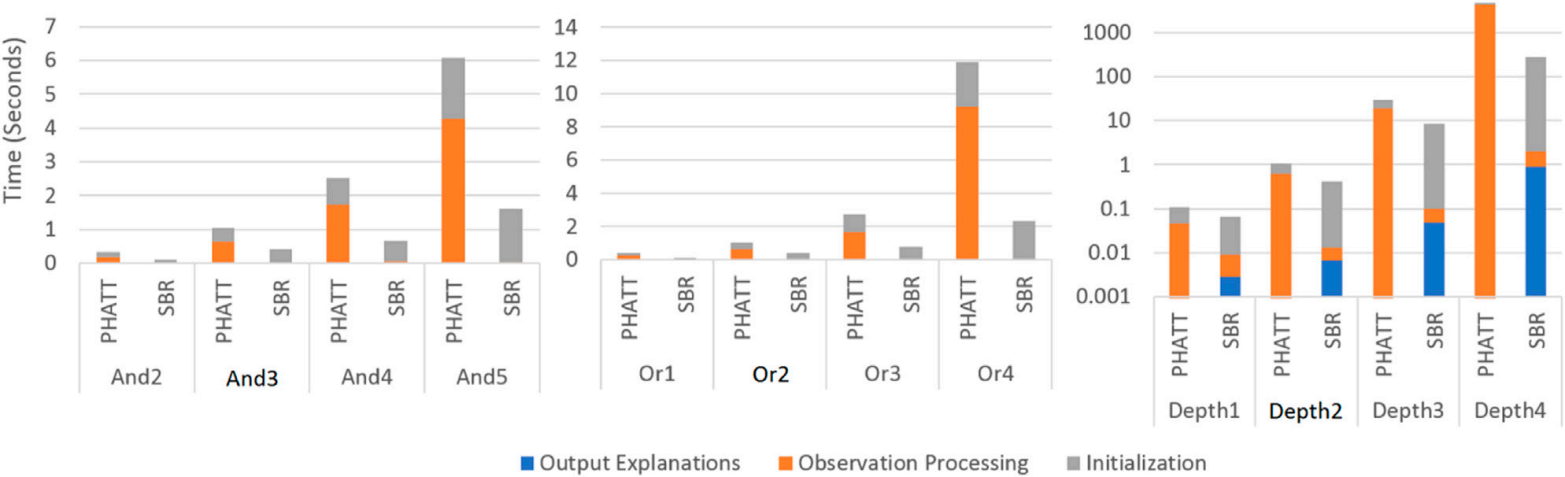
A comparison of execution time by SBR with HSQ and PHATT. In the Depth graph, the y axis is in log scale.

**FIGURE 11:**
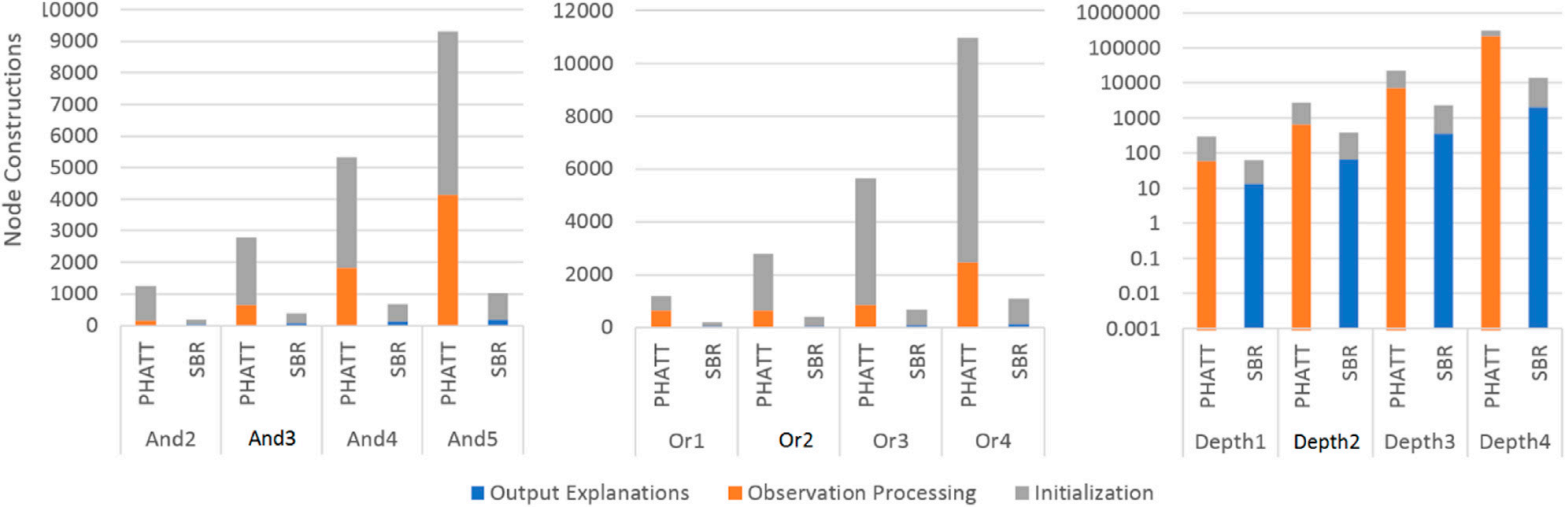
A comparison of space usage by SBR with HSQ and PHATT. In the Depth graph, the y axis is in log scale.

Figures 10, 11 show a comparison of PHATT and SBR (with the HSQ process called once after the last observation) in terms of **node creations** and **time** on 10 domains with different domain values, as described in the previous subsection. The domain parameters that were evaluated are depth, or-branching and and-branching. Each parameter with its permutations is shown in a different graph, while the other parameters are set to the baseline values as described in the previous section. For each combination of domain and algorithm, the bar is divided into three sections:

• Initialization, which is the time (memory) requires to process the domain and create initial structures.
• Observation Processing, the time (memory) requires to process the observations.
• Explanation Creation, the time (memory) requires to combine the information gathered in the previous stages in order to output the final explanations. Notice that in PHATT, this time is always zero, as the observation processing itself requires to construct the explanations.

Figures 10, 11 show respectively the time requires for each algorithm to run and the nodes created (a node is considered a simple structure of constant size, e.g. an action in our representation), where the x-axis is divided into the different domains and algorithms and the y-axis shows the average runtime of 100 instances, counted in seconds. Notice that for the Depth variations, the y-axis is presented in log scale. These two figures show that the depth of the domain is the parameter that has the most impact on the runtime. This is not surprising, as both algorithms produce a number of explanations that is exponential in the depth of the PL, as presented in Section 4.4. PHATT is more affected than SBR from the change in the or-branching. This is probably attributed to the fact that PHATT works in a combinatorial fashion, where it needs to consider every alternative branch to combine into an existing explanation when introduced with a new observation. In all of the domains, SBR pays a penalty for constructing the PL in the initialization phase. However, if we only look at the actual observation processing phase (as the initialization can be processed offline beforehand and the complete explanation construction is not always required), the actual recognition phase in SBR takes an order of magnitude less time than PHATT’s, with smaller usage of space.

Regarding node construction, it is apparent that SBR does not construct nodes in the observation processing phase, but rather in the initialization phase to create the PL and in the explanation output phase (when HSQ is executed). PHATT, on the other hand, builds more compact structures in the initialization phase, but then requires to copy these structures for every new explanation. Thus, most of PHATT’s node construction happens in the observation processing stage. We will now dive deeper to analyze the effects of initialization vs observation processing and explanation construction, by looking at the most demanding domain presented here, with a depth value of 4.

**FIGURE 12:**
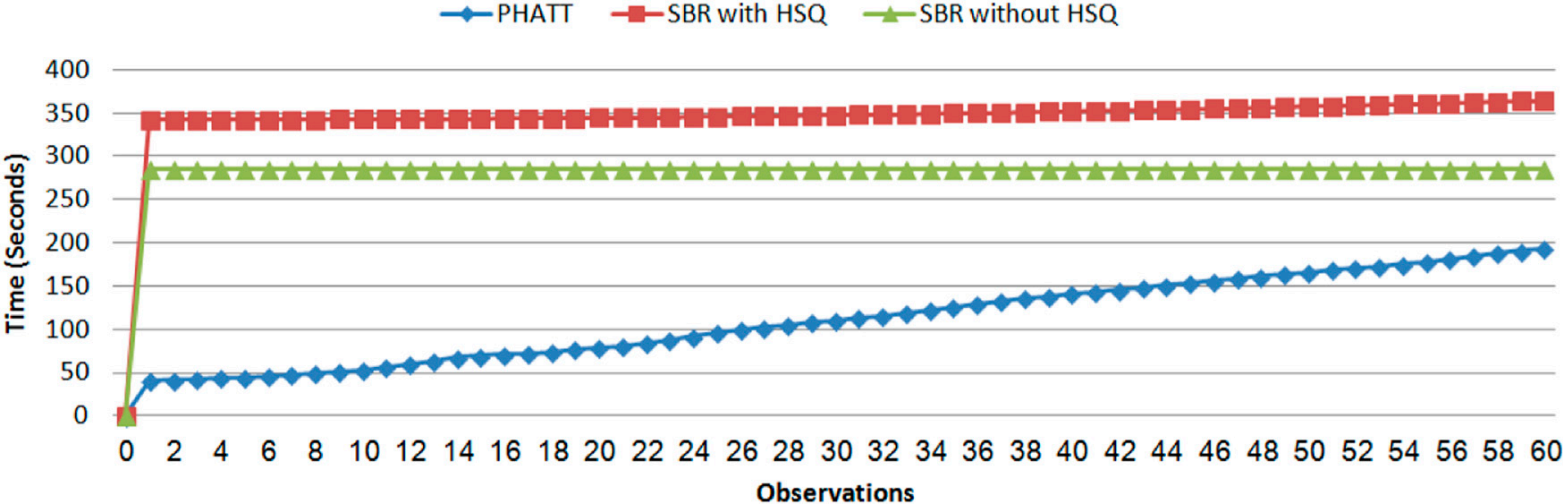
A comparison of time for PHATT, SBR with HSQ after the last observation and SBR without HSQ in the domain with extended depth of 4.

Figure 12 shows the results of the comparison between SBR without explanation creation (the HSQ process), SBR with HSQ and PHATT, in a domain with extended depth value of 4. We remind the reader that even when SBR does not run the HSQ process, it does run the CSQ process for computing a set of hypotheses regarding the current state of the acting agent. As seen in Figure 12, SBR takes up a lot of time to create the map of the complete PL in the initialization phase, but later it does not require any more node constructions (when not applying the HSQ component). PHATT, on the other hand, is relatively light in terms of time in the initialization phase, but then it grows exponentially with the number of observations. The construction of complete explanations from the labeled PL in the HSQ phase, makes SBR’s runtime and node construction exponential, but in a smaller rate than PHATT. Again, the trend of these lines suggests that eventually SBR’s approach will prevail in terms of time-consumption, but this change will happen at a much later stage of the recognition.

So far, Figure 11 showed the benefits of SBR both in terms of runtime and space, and Figure 12 exemplified a complex domain in which SBR’s performance is less impressive. However, it is clear that the observation processing time of SBR is significantly better than PHATT’s in all domains. Next, we wish to show the price that it pays for this speed, which is the type of plans it can capture.

### 5.3 Completeness

As discussed in Section 4 and exemplified in Figure 4, PHATT can inherently capture interleaving plans, while SBR can’t.

**FIGURE 13:**
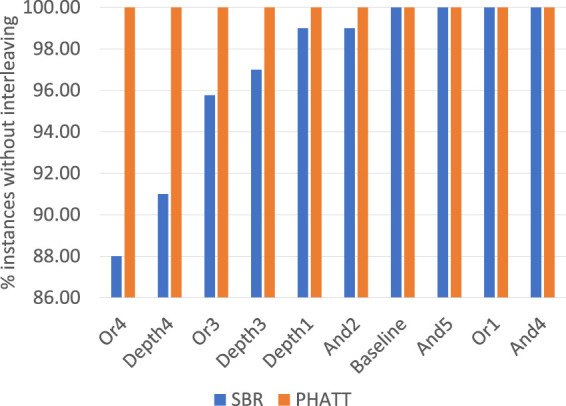
Percentage of instances that each algorithm captures if interleaving is allowed.

Figure 13 shows the percentage of instances in each original domain that does not include interleaving plans. Since PHATT can capture interleaving, its performance is always 100%. As for SBR, it manages to output a complete set of explanations only in the instances that does not include interleaving. As seen in this figure, Or4 is the domain in which interleaving is most prevalent, with 12% of the instances containing explanations with interleaving plans.

## 6 Getting the Best of Both Algorithms

Using the evaluation so far regarding the similarities and differences of the two algorithms, we can now leverage insights that were learned in the process of this work to improve one of the algorithms.

As part of the work on SBR, Avrahami-Zilberbrand and Kaminka presented a component that can match observations to nodes in the PL. In the representation used in this work, each observation can be mapped clearly to a single basic action. However, when SBR was developed, it was required to deal with more complex inputs, where an observation is not necessarily mapped to a basic action, but rather to a complex one. Moreover, there might be some uncertainty regarding the action to map the observation to. There have been previous attempts to deal with this challenge. For example, RESC (REal-time Situated Commitments) (Tambe et al., 1999) handles ambiguous observations by first committing to one interpretation and backtracks when needed. Bui H. H. (2003) presents an algorithm that relies on particle filtering to disabmiguate hypotheses. Sukthankar and Sycara (2011) propose methods to identify team behaviors from traces of agent positions over time. In the original SBR paper, this challenge was address by introducing a Feature Decision Tree (FDT), which efficiently maps observations to matching nodes in the PL. Determining the node that match a set of observation features is efficiently achieved by traversing the FDT top-down, taking branches that correspond to the observed values of features, until a leaf node is reached. While FDT was meant to provide an efficient process to match observations to actions, it also contributed to an improved runtime, by using a predefined dictionary that is used to reach specific nodes in the PL instead of traversing all of the plans for every new observation. We generalized this idea of matching an observation to an entity–either a structure, an action or a set of actions–to make both algorithms more efficient.

In SBR, the matching functionality replaces the function FindNode in **Algorithm 2**, line 4 with a dictionary that is created in the initialization together with the structure of the PL. This dictionary saves the traversal over the complete PL by keeping all nodes with the same label together. In PHATT, the matching process refers to a track kept for previously created generating sets in a dictionary ordered by actions. The matching functionality replaces the call for GenSet in **Algorithm 1**, line 16 and line 26 with an extraction from that dictionary when possible. It is basically makes a memoization of the GenSet process. Importantly, this matching can result in significant improvements both in runtime and space usage, since it can save constructing a new generating set for every observations.

**FIGURE 14:**
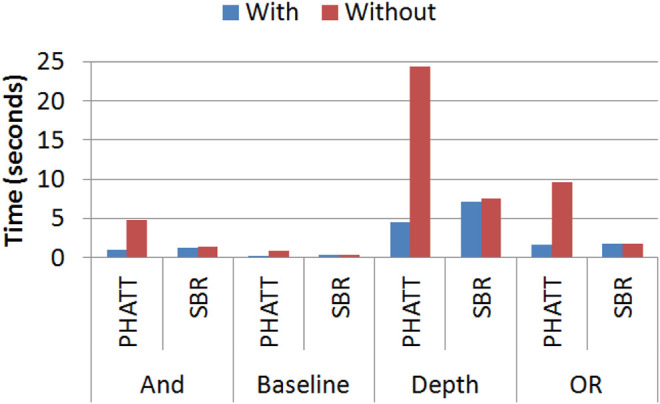
A comparison of runtime (in seconds) with and without using the Matching functionality.

Figure 14 shows the runtime of both PHATT and SBR with and without the added matching functionality on 4 different domains (Baseline, increased And, increased Or, and increased Depth). As seen in the figure, the matching mechanism creates a significant improvement in PHATT’s runtime but not in SBR’s. This is understandable, as the mechanism in PHATT improves its most complex functionality (the observations processing), while in SBR it improves a function that was not its bottleneck in the first place. In complex domains, such as Or4 and Depth3, the improvement of the matching functionality to the PHATT algorithm can reduce the runtime by 80%.

## 7 Conclusion

In this work, we compared and analyzed the differences between two leading PL-based plan recognition algorithms. We conclude by detailing the lessons learned from the use case evaluation above. It contains a list of major points for consideration and a methodical sequence of steps required when comparing new algorithms.

There are a few factors that can effect the outcome when comparing the performance of two algorithms, hence needed to be reasoned about:

Each algorithm focuses its computational efforts in different parts of the recognition process. For example, SBR relies heavily on the construction of the initial PL. Constructing this library takes up a lot of time. However, afterwards, the computation time of the observation processing is very fast as it only needs to label nodes along the created PL. This effect even increases when using the matching ability as discussed below. PHATT, on the other hand, does not need to initialize the complete PL in advance. However, PHATT’s initialization is not memory-free: first it creates the “generating set,” which is the set of all branches that can be grown as a first observation, so even this initialization is not lightweight in terms of memory. We tried to keep these branches using the matching ability, but they are created at some point which takes time and memory. In the observation processing part, PHATT needs to construct new plans for describing new observations, which SBR does not. This in the main computational effort of PHATT, which makes its runtime exponential in the number of observations.

Another difference observed between the algorithms’ processes is the final construction of the outputted explanations: History State Query (HSQ) is a component of the SBR algorithm in which the labeled PL structured is being compiled into a new structure of consistent sequences that can produce explanations. Without it, SBR can still provide an answer regarding the current state of the observed agent, but not the complete explanation. Constructing this new graph has an additional cost, which is exponential in the number of observations in the worst case. However, unlike the explanation construction in PHATT that is an integral part of the algorithm and occurs with every observation, HSQ is called only when the complete explanations are required.

Table 1 summarizes the different computational overload of the two algorithms. Given these points, PHATT is expected to outperform SBR in terms of memory when: 1) the PL is complex enough and 2) we see only a few observations. If 1) does not occur, SBR’s overhead for building all plans is insignificant compared to all possible explanations. If 2) does not occur, PHATT needs to create many explanations, and as the number of explanations is exponential in the number of observations, the initial construction of the whole PL as in SBR is a lighter process in terms of nodes creation. Of course these two measures are connected, and the larger the initial PL is, the more head-start PHATT has to process observations before SBR overcomes it in terms of runtime.

**TABLE 1:** Algorithmic components of PHATT and SBR.

	PHATT	SBR
Initialization	Relatively light (generating set only)	Heavy (constructs the complete PL once)
Observation processing	Heavy (exponential in the number of observations)	Light (matching observations to nodes using a hash function)
Explanations generation	No additional cost (integral part of the processing part)	Heavy (constructs a new structure to find consistent plans)

Using the above domain representation, use case and empirical results, we wish to enable different algorithms to receive the same input and to be evaluated on the same grounds. Before measuring any quantitative value, it is crucial to make sure that the output of the algorithms is the same as well, and that they are trying to solve the same problem. After this stage is done, there are several types of criteria that can be used to evaluate the algorithms in terms of efficiency, robustness and more. The following list is a proposal for the order of the evaluations and tests to perform, as learned from the use case evaluation in this paper.

1) Problem definition Each algorithm focuses on different problems with different features. For example, some algorithms only output the goals of the actor. Others output the goal and a prediction about future actions, but no plan decomposition. In order to compare algorithms, one must first make sure that the algorithms try to solve the same problem. In this work, we chose two of the most comprehensive plan recognition algorithms, that can output the complete plans of the actor.
2) Abilities While we require all compared algorithms to use the same problem definition and output similar results, we know that every algorithm is designed differently, to solve different challenges. This is why we also wish to allow qualitative evaluation of the various abilities of the algorithms. This part of the comparison is also meant to allow each algorithm to highlight its novelty. For example, even given the same input, PHATT and SBR might output different set of explanations if the domain contains interleaving of two plans.
3) Runtime While runtime measurements are practical, it might not be enough for a thorough evaluation. However, as shown in our empirical work, it can provide with some insights when the run times are significantly different between compared algorithms. The runtime measure is divided into the main processes of each algorithm in order to gain insights about the strengths and weaknesses of each algorithm. For example, initialization runtime measures a process that can be executed offline before the actor begins to act, while processing runtime is the actual recognition in real-time.
4) Space Since this measure depends heavily on the implementation, we tried to provide with a more general metric, which is the number of nodes in the plans each algorithm creates. This measure is also good as a sanity check, as there is a lower bound to it, which is the number of nodes in all of the plans that should be outputted. Any algorithm can be evaluated in comparison to this ideal number. This metric should also be divided into two: the total number of nodes created and the average/maximum number of nodes used by the algorithm at a given point in time. The first metric gives us insights about the general space-complexity of the algorithms, while the latter gives us insights about its robustness.

As the aim of this work is to highlight the diversity and variability of plan recognition algorithms by showing one such comparison use-case, we encourage additional standardization efforts that will continue to promote the plan recognition research thrust.

## Data Availability

The datasets presented in this study can be found in online repositories. The names of the repository/repositories and accession number(s) can be found below: https://github.com/ReuthMirsky/Standardization-Project.
